# Medicare Under Age 65 and Medicaid Patients Have Poorer Bowel Preparations: Implications for Recommendations for an Early Repeat Colonoscopy

**DOI:** 10.1371/journal.pone.0155208

**Published:** 2016-05-17

**Authors:** Bryan B. Brimhall, Sam C. Hankins, Vineel Kankanala, Gregory L. Austin

**Affiliations:** 1 Division of Gastroenterology and Hepatology, University of Colorado Denver, Aurora, Colorado, United States of America; 2 Brody School of Medicine, East Carolina University, Greenville, North Carolina, United States of America; 3 Division of Gastroenterology and Hepatology, University of Illinois at Chicago, Chicago, Illinois, United States of America; University Hospital Llandough, UNITED KINGDOM

## Abstract

**Background/Aims:**

Colonoscopy is performed on patients across a broad spectrum of demographic characteristics. These characteristics may aggregate by patient insurance provider and influence bowel preparation quality and the prevalence of adenomas. The purpose of this study was to evaluate the association of insurance status and suboptimal bowel preparation, recommendation for an early repeat colonoscopy due to suboptimal bowel preparation, adenoma detection rate (ADR), and advanced ADR (AADR).

**Methods:**

This is a cohort study of outpatient colonoscopies (n = 3113) at a single academic medical center. Patient insurance status was categorized into five groups: 1) Medicare < 65y; 2) Medicare ≥ 65y; 3) Tricare/VA; 4) Medicaid/Colorado Indigent Care Program (CICP); and 5) commercial insurance. We used multivariable logistic or linear regression modeling to estimate the risks for the association between patient insurance and suboptimal bowel preparation, recommendation for an early repeat colonoscopy due to suboptimal bowel preparation, ADR, and AADR. Models were adjusted for appropriate covariates.

**Results:**

Medicare patients < 65y (OR 4.91; 95% CI: 3.25–7.43) and Medicaid/CICP patients (OR 4.23; 95% CI: 2.65–7.65) were more likely to have a suboptimal preparation compared to commercial insurance patients. Medicare patients < 65y (OR 5.58; 95% CI: 2.85–10.92) and Medicaid/CICP patients (OR 3.64; CI: 1.60–8.28) were more likely to receive a recommendation for an early repeat colonoscopy compared to commercial insurance patients. Medicare patients < 65y had a significantly higher adjusted ADR (OR 1.50; 95% CI: 1.03–2.18) and adjusted AADR (OR 1.99; 95% CI: 1.15–3.44) compared to commercial insurance patients.

**Conclusions:**

Understanding the reasons for the higher rate of a suboptimal bowel preparation in Medicare < 65y and Medicaid/CICP patients and reducing this rate is critical to improving colonoscopy outcomes and reducing healthcare costs in these populations.

## Introduction

Colorectal cancer (CRC) is the third leading cause of cancer-related mortality in the United States in both men and women, and is second overall [1]. Colonoscopy is the most commonly utilized method for CRC screening in the U.S., in part because pre-cancerous adenomas in the colon can be detected and removed in a single procedure. However, maximal benefit from colonoscopy requires an optimal bowel preparation, as inadequate bowel preparation has been shown to decrease the adenoma detection rate (ADR) [2]. An inadequate bowel preparation can also result in a recommendation for a shorter screening or surveillance interval than would otherwise be clinically indicated, leading to increased healthcare costs.

Several factors have been associated with an inadequate or suboptimal bowel preparation. This includes a failure to understand and follow preparation instructions, low socioeconomic status, need for an interpreter, comorbid conditions, diverticulosis, and medications associated with constipation [3–7]. There have been a few reports evaluating the quality of the bowel preparation based on the insurance provider of the patient. Medicaid insurance status has been associated with an increased likelihood of having a suboptimal bowel preparation [3, 8, 9]. Conversely, patients with Medicare and commercial insurance have been reported as being more likely to have adequate bowel preparations [9]. However, these studies did not assess other important outcomes, including whether inadequate or suboptimal preparations led to recommendations for a screening or surveillance interval that was shorter than would otherwise be clinically indicated, the ADR, the advanced adenoma detection rate (AADR), and procedure time characteristics. Furthermore, outcomes for the subset of Medicare patients under the age of 65y have not been reported.

The purpose of this study was to assess whether an easily identifiable patient characteristic such as patient insurance provider was associated with a suboptimal bowel preparation and a subsequent recommendation for a shorter interval before the next recommended colonoscopy (for screening or surveillance) than would otherwise have been recommended. Secondary outcomes included the ADR, AADR, and procedure time characteristics.

## Methods

### Study Population

This is a retrospective cohort study of all outpatient colonoscopies performed at the University of Colorado Hospital (UCH) between July 2011 and October 2012. This study was approved by the Colorado Multiple Institutional Review Board (COMIRB). All patients were given the same printed instructions regarding the administration of the bowel preparation. Each bowel preparation solution was given in a split-dose schedule (half of the preparation the night before the procedure and the other half the morning of the procedure), as this method has proven to be more efficacious with respect to bowel preparation quality [10]. Patients with a history of inflammatory bowel disease (n = 127) and a personal history of CRC (n = 74) were excluded. Patients with an indication for a fecal transplant secondary to clostridium difficile colitis were also excluded (n = 4). Inpatient colonoscopies were also excluded. All procedures were performed with high-definition Olympus^®^ colonoscopes. Because this was a retrospective cohort study, neither patients nor providers knew they were being studied. Additionally, because this was a retrospective study, consent from the patients whose procedures were included in this study was not possible and a waiver of consent was obtained from COMIRB.

### Data Collection

The UCH electronic medical record (Epic; Verona, WI) and the endoscopy reporting database (Provation^®^; Minneapolis, MN) were used to extract covariate and outcome data pertinent to this study. Variables that were extracted: patient insurance provider, age, gender, indication for colonoscopy (diagnostic or screening/surveillance), fellow involvement, need for an interpreter, having a chronic pain diagnosis, outpatient use of opiate medications, and the specific bowel preparation used (Moviprep^®^ or Colyte^®^). We extracted the total number of polyps and the size of the largest polyp for each patient and included these in the multivariable models assessing procedure time outcomes as these characteristics had a significant effect on those outcomes. Additionally, the individual attending endoscopist (n = 8) was recorded. Patient insurance status was categorized into five groups according to insurance provider at the time of the colonoscopy: Medicare patients under 65 years of age (n = 174), Medicare patients 65 years and older (n = 814), Tricare/VA Champus (n = 634), Medicaid/Colorado Indigent Care Program (CICP) (n = 168), and commercial/private insurance (n = 1323). Patients with Medicaid or CICP were grouped together because patients in these groups migrate between the two programs. CICP provides discounted health care services to low-income individuals and families in Colorado [11].

The primary outcomes investigated were the percentage of patients with a suboptimal bowel preparation quality and a recommendation for an early repeat colonoscopy due to the suboptimal bowel preparation. ADR, AADR, insertion time, withdrawal time, and total procedure time were secondary outcomes. Bowel preparation was rated by the attending endoscopist according to the modified Aronchick scale and recorded in the endoscopy report [12]. This scale uses the following criteria: Poor/Inadequate—poor prep quality, exam still completed, feces and/or turbid fluid make prep unreliable and less than 90% of the mucosa is visualized; Fair—moderate amount of stool that may be adequately cleared via suctioning to permit adequate evaluation, over 90% of the mucosa can be visualized; Good—some turbid fluid without feces, no interference with exam, more than 90% of mucosa visualized; Excellent—small amount of clear liquid with over 95% of the mucosa visualized [12]. Prep quality was dichotomized into optimal (good or excellent) and suboptimal (fair, poor, or inadequate).

Total procedure time was calculated from the time stamps in Provation^®^ that identify “Scope In” as the start of the procedure and “Scope Out” as the completion of the procedure. Insertion time was calculated from the time stamps that identify “Scope In” and “Cecum Reached”. Withdrawal time was calculated from the time stamps that identify “Cecum Reached” and “Scope Out”. The ADR was calculated as the percentage of patients in each group who had at least one adenoma (and included those with sessile serrated polyps). The AADR was calculated as the percentage of patients in each group who had at least one advanced adenoma on the basis of size (any adenoma or sessile serrate polyp ≥ 10mm) or histology (adenomas containing villous histology or high-grade dysplasia regardless of size or a sessile serrated polyp with dysplasia). For patients with a suboptimal preparation, an early repeat colonoscopy was defined when the interval that was recommended was clearly not indicated based on the findings of the colonoscopy, a patient’s family history, or a patient’s prior history of adenomatous polyps or cancer.

### Statistical Analysis

All data were analyzed using STATA 10.0 statistical software (StataCorp, College Station, Texas). Demographic and baseline characteristics for the five groups were compared using the analysis of variance (ANOVA) and the chi-square test. We used multivariable logistic or linear regression modeling to estimate the risks for the association between patient insurance and all outcomes. The following covariates were included in the multivariable logistic regression models for suboptimal preparation quality, recommendation for an early repeat colonoscopy, ADR, and AADR: age, gender, indication for colonoscopy (diagnostic versus screening/surveillance), the specific bowel preparation (Moviprep^®^ or Colyte^®^), fellow involvement, need for an interpreter, a chronic pain diagnosis, and outpatient use of opiate medications. The following covariates were included in the multivariable linear regression models for insertion, withdrawal, and total procedure time outcomes: age, gender, indication for colonoscopy (diagnostic versus screening/surveillance), the specific bowel preparation (Moviprep^®^ or Colyte^®^), fellow involvement, need for an interpreter, a chronic pain diagnosis, outpatient use of opiate medications, total number of polyps resected, and size of the largest polyp resected. Because of differences in prep quality ratings, the detection of adenomas, and procedure time outcomes between endoscopists, appropriate dummy variables for each endoscopist were included in all multivariable models. Unless specifically stated, those with commercial insurance were chosen as the referent category as this was the largest group in our study and is the most reflective of a general population.

Because there were differences in the covariates between the groups, we calculated adjusted percentages (for the outcomes of suboptimal preparation quality, recommendation for an early repeat colonoscopy, ADR, and AADR) and adjusted means (for procedure time outcomes) using the “predxcat” command in STATA. To examine whether there was effect modification of the relationship between a diagnostic indication for colonoscopy and insurance, we created an interaction term for indication-insurance. To assess for effect modification by gender and fellow involvement in the colonoscopy (with insurance), we also created an interaction term for gender and insurance and an interaction term for fellow involvement in the colonoscopy and insurance.

## Results

As expected (Table 1), there were significant differences in demographics among the five groups of patients. Medicare patients > 65y were the oldest age group (p<0.001). More patients in the Medicare < 65y and Medicaid/CICP groups underwent colonoscopy for diagnostic purposes in comparison with the other groups (p<0.001). Commercial insurance, Tricare/VA, and Medicare > 65y patients were more likely to have used Moviprep^®^ compared to the Medicare < 65y and Medicaid/CICP patients (p<0.001). Medicaid/CICP patients were more likely to require interpreter services than the other groups (p<0.001). Medicare patients < 65y were more likely to have a chronic pain diagnosis than patients in other groups and to have an active outpatient prescription for opioids (both p<0.001). Gender was evenly distributed among the groups (p = 0.34).

**Table 1:** Patient Demographic and Clinical Characteristics by Insurance Provider.

	Commercial (n = 1323)	Medicare Under 65 (n = 174)	Medicare Over 65 (n = 814)	Tricare/VA Champus (n = 634)	Medicaid/CICP (n = 168)	p-value
Age (± S.D.), y	53.5 ± 9.8	52.6 ± 8.4	71.3 ± 4.9	55.2 ± 7.8	51.9 ± 12.5	<0.001
Gender (% Women)	53.1	58.1	51.7	56	55.9	0.344
Indication (% Diagnostic)	24.1	40.2	19.2	17.5	49.4	<0.001
Prep Type (% Moviprep^®^)	63.4	46.6	58.2	57.6	44.1	<0.001
Fellow Involvement (%)	10.8	28.7	15.6	14.2	46.4	<0.001
Required Interpreter (%)	2.0	2.3	4.9	0.8	25.3	<0.001
Chronic Pain Diagnosis (%)	21.1	51.5	29.7	20.3	23.5	<0.001
Opioid Use (%)	14.3	52	23	18.1	46.4	<0.001
Mean Number of Polyps (± S.D.)	0.9 ± 1.5	1.3 ± 2.1	1.5 ± 2.0	1.0 ± 1.7	1.0 ± 1.6	<0.001
Mean Size of Largest Polyp (± S.D.), mm	6.4 ± 5.7	8.2 ± 11.4	7.0 ± 6.8	5.6 ± 4.4	7.3 ± 6.9	0.004

### Suboptimal Bowel Preparation

**Fig 1:**
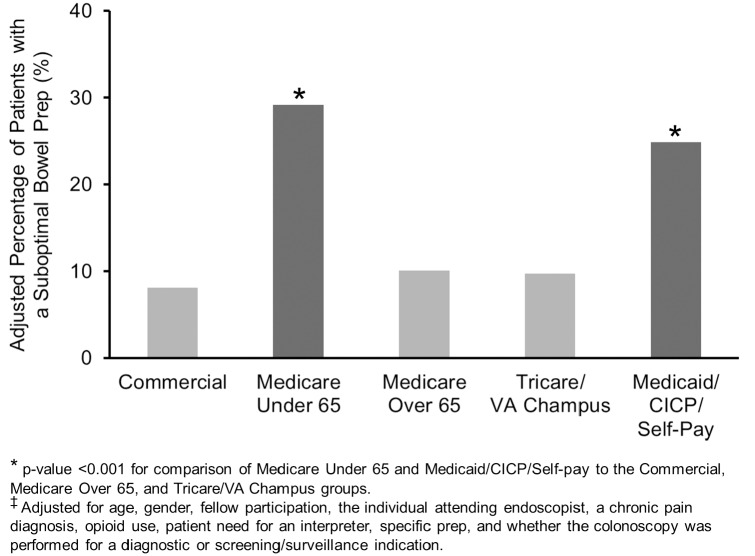
A Higher Percentage^‡^ of Medicare Patients Under 65 and Medicaid/CICP/Self-Pay Patients had a Suboptimal Bowel Prep.

Use of Moviprep^®^, need for an interpreter, and a chronic pain diagnosis were associated with a suboptimal bowel preparation (Table 2). The adjusted percentage (controlled for the potential confounders) with a suboptimal preparation was 29.2% for Medicare patients < 65y and was 24.9% for Medicaid patients. The adjusted percentage with a suboptimal preparation was 10% or less for the other 3 groups. Medicare patients < 65y were more likely to have a suboptimal bowel preparation (Fig 1) when compared to commercial insurance (OR 4.91), Medicare > 65y (OR 4.08), and Tricare/VA (OR 4.21) patients [all p<0.001]. Medicaid/CICP patients were also more likely to have a suboptimal bowel preparation in comparison to commercial insurance (OR 4.23), Medicare > 65y (OR 3.51), and Tricare/VA (OR 3.62) patients [all p<0.001]. The rate of suboptimal bowel preparation for Medicare < 65y patients compared to Medicaid/CICP patients was similar (p = 0.57). Commercial insurance, Medicare > 65y, and Tricare/VA patients had similar rates of having a suboptimal bowel preparation when compared to each other (all p>0.32). There was no effect modification of the relationship between insurance and a suboptimal bowel preparation by gender, a diagnostic (versus screening/surveillance) colonoscopy indication, or fellow participation (Table 3).

**Table 2:** Association of Covariates with Outcomes in Multivariable Analysis.

**Suboptimal Prep Quality**	Odds Ratio	95% CI	P>z
Age (per year)	1.01	(1.00–1.03)	0.087
Male Gender	1.00	(0.80–1.26)	0.968
Diagnostic Indication	0.90	(0.68–1.20)	0.480
Moviprep^®^	1.36	(1.06–1.76)	0.017
Fellow Participation	0.98	(0.71–1.34)	0.880
Interpreter Needed	0.47	(0.24–0.93)	0.030
Chronic Pain Diagnosis	1.63	(1.26–2.11)	<0.001
Opioid Use	0.93	(0.70–1.25)	0.637
**Early Repeat Colonoscopy Recommendation**	Odds Ratio	95% CI	P>z
Age (per year)	1.02	(1.00–1.05)	0.077
Male Gender	0.87	(0.57–1.32)	0.515
Diagnostic Indication	0.96	(0.58–1.58)	0.875
Moviprep^®^	1.16	(0.72–1.88)	0.533
Fellow Participation	1.33	(0.75–2.36)	0.327
Interpreter Needed	0.68	(0.23–2.04)	0.490
Chronic Pain Diagnosis	1.35	(0.83–2.18)	0.223
Opioid Use	0.93	(0.54–1.57)	0.774
**Adenoma Detection Rate**	Odds Ratio	95% CI	P>z
Age (per year)	1.05	(1.03–1.06)	<0.001
Male Gender	1.26	(1.08–1.48)	0.004
Diagnostic Indication	0.58	(0.47–0.72)	<0.001
Moviprep^®^	0.93	(0.78–1.11)	0.428
Fellow Participation	1.64	(1.30–2.06)	<0.001
Interpreter Needed	1.27	(0.83–1.93)	0.271
Chronic Pain Diagnosis	0.71	(0.58–0.87)	0.001
Opioid Use	1.24	(0.99–1.54)	0.057
**Advanced Adenoma Detection Rate**	Odds Ratio	95% CI	P>z
Age (per year)	1.02	(1.01–1.04)	0.007
Male Gender	1.20	(0.92–1.57)	0.179
Diagnostic Indication	1.11	(0.80–1.55)	0.537
Moviprep^®^	1.18	(0.87–1.62)	0.291
Fellow Participation	1.13	(0.76–1.69)	0.544
Interpreter Needed	1.22	(0.63–2.34)	0.554
Chronic Pain Diagnosis	0.59	(0.41–0.84)	0.003
Opioid Use	1.43	(1.01–2.02)	0.045

**Table 3:** Multivariable Analyses of Suboptimal Bowel Preparation, Recommendation for an Early Repeat Colonoscopy, Adenoma Detection Rate, and Advanced Adenoma Detection Rate Stratified by Gender, Colonoscopy Indication, and Fellow Involvement.

**Suboptimal Bowel Preparation**	**Interaction p-value**^1^	**Subgroup**	**Odds Ratio**	**95% CI**	**p-value**
		**Women**^2^			
		Medicare Under 65	4.66	(2.69–8.10)	<0.001
		Medicaid/CICP	4.51	(2.44–8.33)	<0.001
Gender-Insurance Interaction	0.511				
		**Men**^2^			
		Medicare Under 65	5.34	(2.83–10.06)	<0.001
		Medicaid/CICP	3.41	(1.62–7.17)	0.001
		**Screening/Surveillance**^3^			
		Medicare Under 65	5.02	(3.01–8.35)	<0.001
		Medicaid/CICP	3.89	(2.07–7.30)	<0.001
Colonoscopy Indication-Insurance Interaction	0.918				
		**Diagnostic**^3^			
		Medicare Under 65	4.83	(2.30–10.16)	<0.001
		Medicaid/CICP	4.75	(2.25–10.01)	<0.001
		**No Fellow**^4^			
		Medicare Under 65	4.74	(2.94–7.64)	<0.001
		Medicaid/CICP	3.79	(2.06–6.96)	<0.001
Fellow Involvement-Insurance Interaction	0.887				
		**With Fellow**^4^			
		Medicare Under 65	5.28	(2.10–13.25)	<0.001
		Medicaid/CICP	3.92	(1.63–9.43)	0.002
**Recommendation for Early Repeat Colonoscopy**	**Interaction p-value**^1^	**Subgroup**	**Odds Ratio**	**95% CI**	**p-value**
		**Women**^2^			
		Medicare Under 65	5.58	(2.25–13.82)	<0.001
		Medicaid/CICP	4.16	(1.47–11.76)	0.007
Gender-Insurance Interaction	0.670				
		**Men**^2^			
		Medicare Under 65	6.50	(2.35–18.01)	<0.001
		Medicaid/CICP	2.46	(0.60–10.07)	0.212
		**Screening/Surveillance**^3^			
		Medicare Under 65	5.10	(2.28–11.40)	<0.001
		Medicaid/CICP	3.01	(1.05–8.65)	0.041
Colonoscopy Indication-Insurance Interaction	0.834				
		**Diagnostic**^3^			
		Medicare Under 65	8.28	(2.15–31.84)	0.002
		Medicaid/CICP	7.36	(1.79–30.23)	0.006
		**No Fellow**^4^			
		Medicare Under 65	5.75	(2.64–12.49)	<0.001
		Medicaid/CICP	3.99	(1.44–11.04)	0.008
Fellow Involvement-Insurance Interaction	0.410				
		**With Fellow**^4^			
		Medicare Under 65	5.02	(1.08–23.33)	0.040
		Medicaid/CICP	2.17	(0.47–10.09)	0.322
**Adenoma Detection Rate**	**Interaction p-value**^1^	**Subgroup**	**Odds Ratio**	**95% CI**	**p-value**
		**Women**^2^			
		Medicare Under 65	1.81	(1.09–3.00)	0.021
		Medicaid/CICP	0.79	(0.44–1.43)	0.445
Gender-Insurance Interaction	0.289				
		**Men**^2^			
		Medicare Under 65	1.21	(0.69–2.13)	0.501
		Medicaid/CICP	0.91	(0.50–1.69)	0.774
		**Screening/Surveillance**^3^			
		Medicare Under 65	1.29	(0.82–2.02)	0.274
		Medicaid/CICP	0.88	(0.52–1.50)	0.648
Colonoscopy Indication-Insurance Interaction	0.140				
		**Diagnostic**^3^			
		Medicare Under 65	1.91	(0.96–3.80)	0.066
		Medicaid/CICP	0.81	(0.38–1.71)	0.579
		**No Fellow**^4^			
		Medicare Under 65	1.54	(0.99–2.41)	0.056
		Medicaid/CICP	0.76	(0.41–1.40)	0.380
Fellow Involvement-Insurance Interaction	0.608				
		**With Fellow**^4^			
		Medicare Under 65	1.13	(0.54–2.37)	0.747
		Medicaid/CICP	0.79	(0.40–1.54)	0.483
**Advanced Adenoma Detection Rate**	**Interaction p-value**^1^	**Subgroup**	**Odds Ratio**	**95% CI**	**p-value**
		**Women**^2^			
		Medicare Under 65	2.07	(1.01–4.27)	0.048
		Medicaid/CICP	0.73	(0.26–2.05)	0.552
Gender-Insurance Interaction	0.338				
		**Men**^2^			
		Medicare Under 65	1.81	(0.75–4.37)	0.184
		Medicaid/CICP	1.25	(0.50–3.16)	0.636
		**Screening/Surveillance**^3^			
		Medicare Under 65	1.44	(0.68–3.06)	0.344
		Medicaid/CICP	0.84	(0.31–2.30)	0.738
Colonoscopy Indication-Insurance Interaction	0.545				
		**Diagnostic**^3^			
		Medicare Under 65	2.58	(1.07–6.21)	0.034
		Medicaid/CICP	1.12	(0.41–3.01)	0.827
		**No Fellow**^4^			
		Medicare Under 65	1.43	(0.72–2.86)	0.309
		Medicaid/CICP	0.71	(0.26–1.90)	0.495
Fellow Involvement-Insurance Interaction	0.768				
		**With Fellow**^4^			
		Medicare Under 65	4.41	(1.46–13.33)	0.009
		Medicaid/CICP	1.62	(0.50–5.21)	0.417

Note: All Odds Ratios are in comparison to the commercial insurance group.

^1^Odds ratios are adjusted for age, gender, fellow participation, a chronic pain diagnosis, opioid use, patient need for an interpreter, the specific prep, the individual attending endoscopist, and whether the colonoscopy was performed for a diagnostic indication.

^2^Odds ratios are adjusted for age, fellow participation, a chronic pain diagnosis, opioid use, patient need for an interpreter, the specific prep, the individual attending endoscopist, and whether the colonoscopy was performed for a diagnostic indication.

^3^Odds ratios are adjusted for age, gender, fellow participation, a chronic pain diagnosis, opioid use, patient need for an interpreter, the specific prep, and the individual attending endoscopist.

^4^Odds ratios are adjusted for age, gender, a chronic pain diagnosis, opioid use, patient need for an interpreter, the specific prep, the individual attending endoscopist, and whether the colonoscopy was performed for a diagnostic indication.

### Recommendation for an Early Repeat Colonoscopy

**Fig 2:**
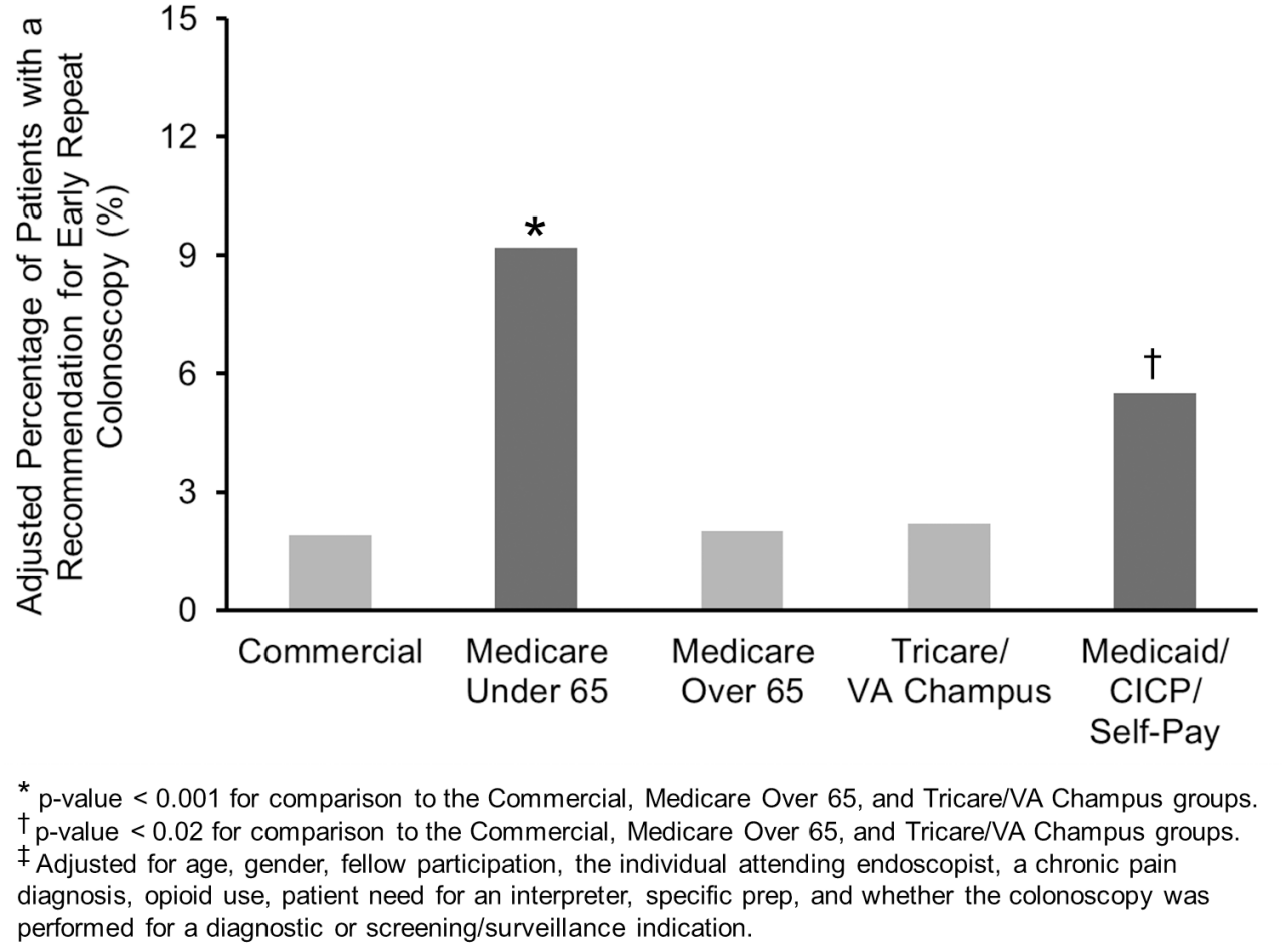
A Higher Percentage^‡^ of Medicare Patients Under 65 and Medicaid/CICP/Self-Pay Patients Received a Recommendation for an Early Repeat Colonoscopy.

None of the covariates (in Table 2) were associated with a recommendation for an early repeat colonoscopy. As would be expected, the results for patients receiving a recommendation for an early repeat colonoscopy were quite similar to the results for patients having a suboptimal bowel preparation. Medicare patients < 65y were more likely to receive a recommendation for an early repeat colonoscopy (Fig 2) when compared to commercial insurance (OR 5.58), Medicare > 65y (OR 5.50), and Tricare/VA patients (OR 4.92) [all p<0.001]. Medicaid/CICP patients were also more likely to receive a recommendation for an early repeat colonoscopy when compared to commercial insurance (OR 3.64), Medicare > 65y (OR 3.59), and Tricare/VA patients (OR 3.21) [all p<0.011]. There was a similar rate of receiving a recommendation for an early repeat colonoscopy between Medicare < 65y and Medicaid/CICP patients (p = 0.32). Commercial insurance, Medicare > 65y, and Tricare/VA patients were equally likely to receive a recommendation for an early repeat colonoscopy when compared to each other (all p>0.71). There was no effect modification of the relationship between insurance and receiving a recommendation for an early repeat colonoscopy by gender, a diagnostic (versus screening/surveillance) colonoscopy indication, or fellow participation (Table 3). The (weighted) percentage of Commercial, Medicare > 65, and Tricare insurance with a suboptimal bowel preparation and recommendation for an early repeat colonoscopy was 9.1% and 2.0%, respectively, compared to the weighted respective percentages of 27.1% and 7.4% for the Medicare <65y and Medicaid patients. An intervention for Medicare <65y and Medicaid patients that could achieve the same rate for a suboptimal bowel preparation (and subsequent recommendation for an early repeat colonoscopy) compared to all others would result in 180 fewer suboptimal bowel preparations and 54 fewer early repeat colonoscopies for every 1000 colonoscopies performed in these patients.

### Adenoma Detection Rate (ADR) and Advanced Adenoma Detection Rate (AADR)

**Fig 3:**
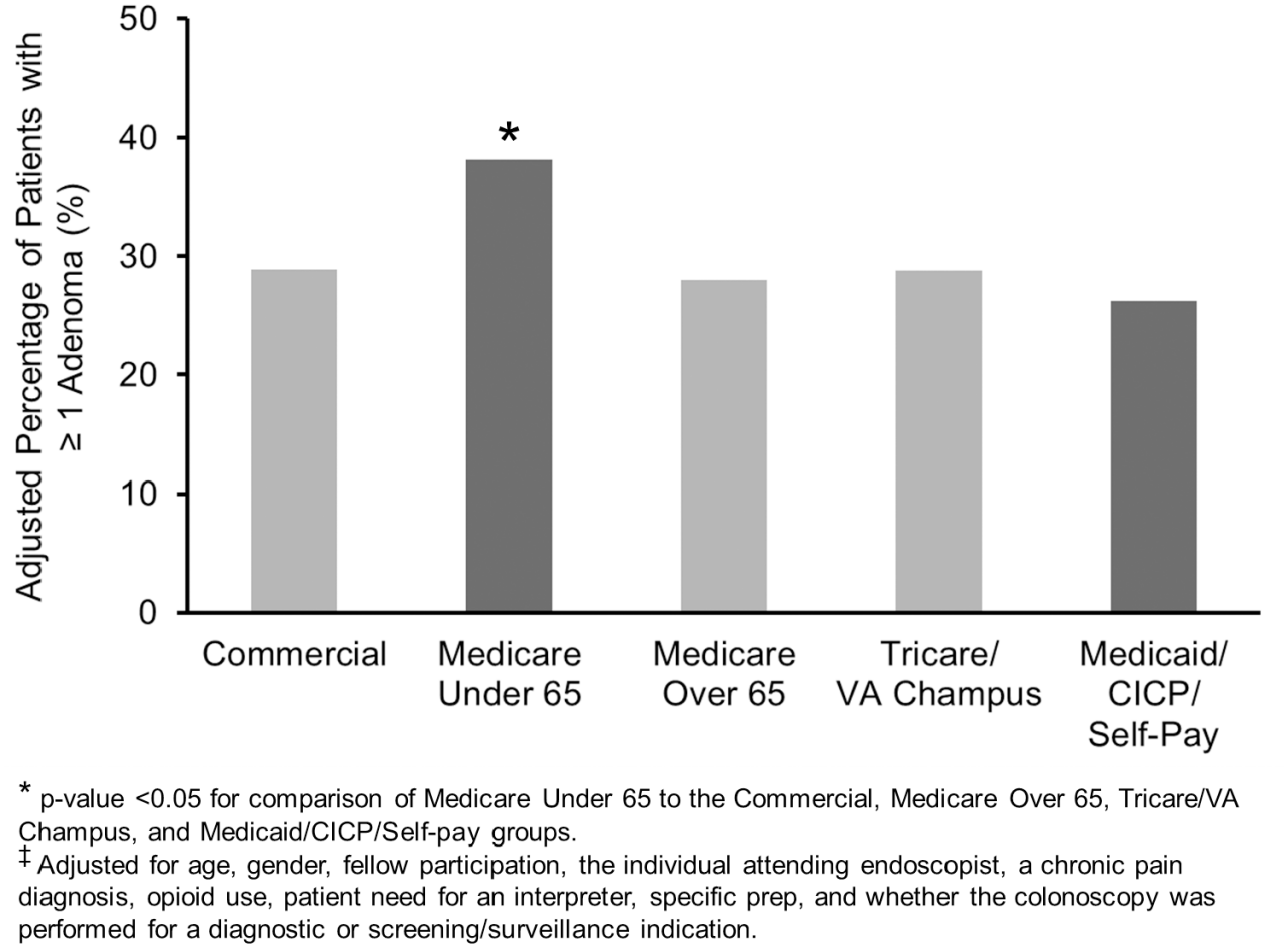
A Higher Percentage^‡^ of Medicare Under 65 Patients had at least One Adenoma.

**Fig 4:**
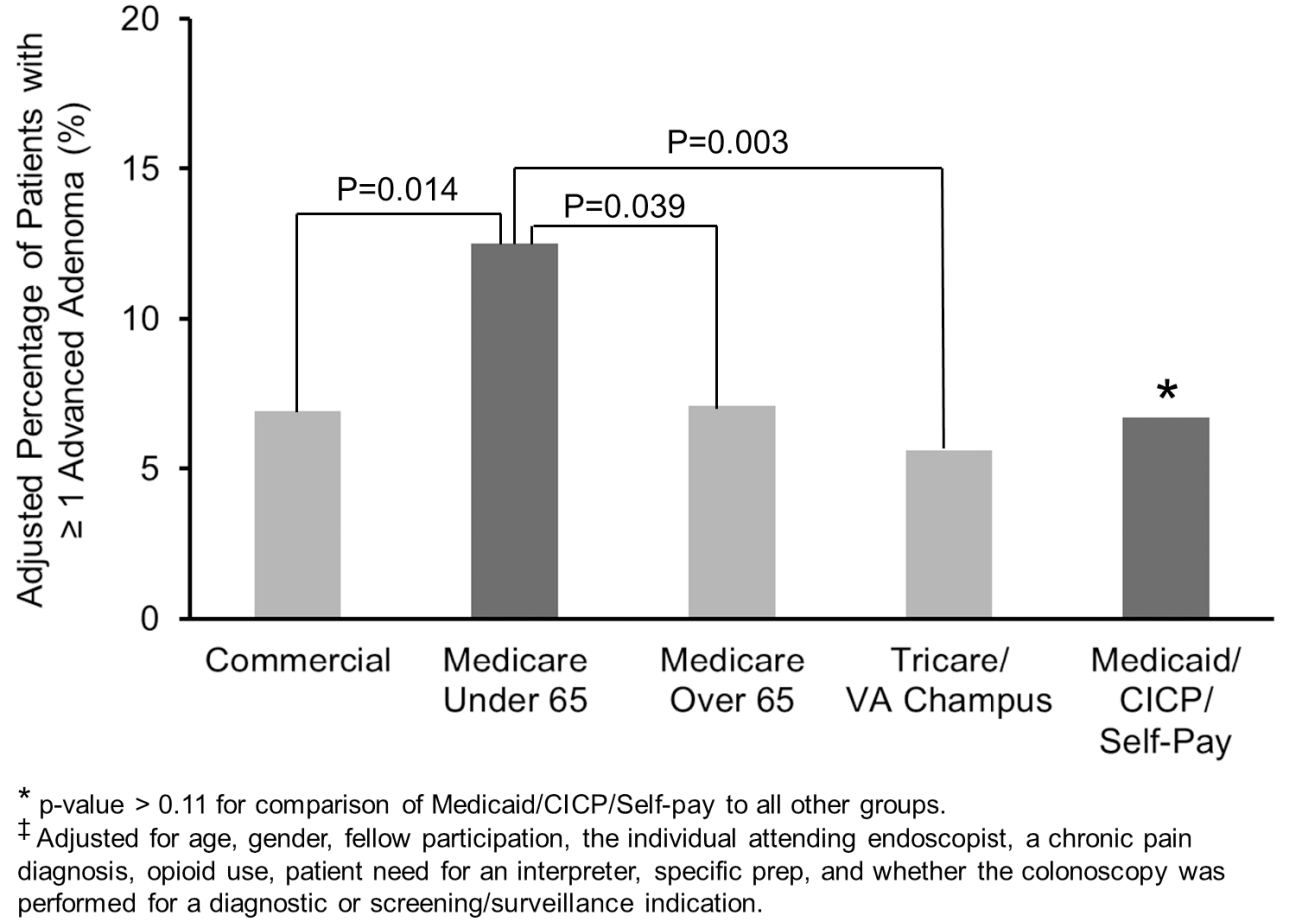
A Higher Percentage^‡^ of Medicare Under 65 Patients had at Least One Advanced Adenoma.

As expected, increasing age and male gender were associated with an increase in the odds of having an adenoma or advanced adenoma (Table 2). A diagnostic indication was associated with reduced odds of having an adenoma but was not associated with having an advanced adenoma. Interestingly, a chronic pain diagnosis was associated decreased odds of having an adenoma and an advanced adenoma, while opioid use was associated a borderline significant increase in the odds of having an adenoma and an advanced adenoma. The unadjusted ADR was 26.4% for commercial insurance, 33.3% for Medicare < 65y, 41.9% for Medicare > 65y, 28.1% for Tricare/VA Champus, and 27.4% for Medicaid/CICP. The unadjusted AADR was 6.8% for commercial insurance, 12.1% for Medicare < 65y, 10.0% for Medicare > 65y, 5.5% for Tricare/VA Champus, and 8.4% for Medicaid/CICP. Because of the obvious differences in age for the Medicare > 65y group compared to all the other groups and the strong positive association between age and the ADR (p<0.001) and AADR (p = 0.007), we calculated an adjusted ADR and AADR for all 5 patients groups (adjusting for the potential confounders listed in the Methods section, including age). The adjusted ADR was 28.9% for commercial insurance, 38.2% for Medicare < 65y, 28.0% for Medicare > 65y, 28.8% for Tricare/VA Champus, and 26.2% for Medicaid/CICP. The adjusted AADR was 6.9% for commercial insurance, 12.5% for Medicare < 65y, 7.1% for Medicare > 65y, 5.6% for Tricare/VA Champus, and 6.7% for Medicaid/CICP. The adjusted ADR was significantly higher for Medicare patients < 65y (Fig 3) compared to commercial insurance (OR 1.50), Medicare > 65y (OR 1.59), Tricare/VA (OR 1.51), and Medicaid/CICP patients (OR 1.69) [all p<0.044]. The adjusted ADR was similar when the commercial insurance, Medicare > 65y, Tricare/VA, and Medicaid/CICP patients were compared to each other (all p>0.57). The adjusted AADR was significantly higher for Medicare patients < 65y (Fig 4) compared to commercial insurance (OR 1.99), Medicare > 65y (OR 1.94), and Tricare/VA (OR 2.49) patients [all p<0.039]. There was a non-significant increase in the adjusted AADR in Medicare < 65y patients compared to Medicaid/CICP patients (OR 1.89; p = 0.11). The adjusted AADR was similar when the commercial insurance, Medicare > 65y, Tricare/VA, and Medicaid/CICP patients were compared to each other (all p>0.28). There was no effect modification of the relationship between insurance and having an adenoma or an advanced adenoma by gender, a diagnostic (versus screening/surveillance) colonoscopy indication, or fellow participation (Table 3).

### Procedure Times

Consistent with the increased probability of having a suboptimal preparation, the mean (± S.E.) total procedure time was significantly longer for Medicaid/CICP patients by 3.54 ± 1.05 minutes (p = 0.001) and for Medicare patients < 65y by 1.99 ± 1.02 minutes (p = 0.05) when compared to commercial insurance patients. Interestingly, the longer procedure time for Medicare < 65y patients was mostly explained by a longer insertion time (of 1.54 ± 0.63 minutes; p = 0.016) as there was not a significant difference in withdrawal time (p = 0.47). Conversely, the longer total procedure time for Medicaid/CICP patients was explained by both a longer insertion time (of 1.41 ± 0.66 minutes; p = 0.033) and a longer withdrawal time (of 3.27 ± 0.80 minutes; p<0.001). There were not any differences in total procedure, insertion, or withdrawal time for Medicare > 65y and Tricare/VA patients compared to commercial insurance patients (all p>0.46).

## Discussion

The primary aims of this study were to identify whether the insurance provider of patients undergoing an outpatient colonoscopy was predictive of having a suboptimal bowel preparation resulting in a subsequent recommendation for an earlier repeat screening or surveillance colonoscopy than would otherwise be clinically indicated. The results of this study demonstrate that patients under the age of 65 with Medicare and patients covered by Medicaid/CICP in Colorado were about 4 times as likely to have a suboptimal bowel preparation and, therefore, receive a recommendation for an early repeat colonoscopy. Our findings are novel in that the increase in suboptimal bowel preparation (and recommendation for an early repeat colonoscopy) have not been previously reported or studied in Medicare < 65y patients. Our results for Medicaid patients are consistent with previous reports that Medicaid patients are more likely to have a suboptimal bowel preparation [3, 8]. The importance of a suboptimal bowel preparation in Medicare patients < 65y is amplified by the additional finding that these patients were more likely to have adenomas and advanced adenomas. It is unclear why Medicare < 65y patients were more likely to have adenomas and advanced adenomas despite being more likely to have a suboptimal bowel preparation. The magnitude of the increased ADR and AADR for Medicare < 65y patients would have been even greater if we had further adjusted for having a suboptimal bowel preparation in our multivariable models for these outcomes.

Medicare patients < 65y represent a unique patient population. To qualify for Medicare under the age of 65y, an individual must meet criteria for disability [13]. Patients who meet these requirements may be more likely to have comorbid medical conditions that could result in a suboptimal bowel preparation for patients undergoing colonoscopy [14]. One study examining the prescribing habits of primary-care physicians over a ten-year period from 1992 to 2001 showed that Medicare patients were twice as likely to receive opioids compared to non-Medicare patients, though no distinction was made based on age [15]. However, opioid use was not the explanation for our findings as we controlled for a chronic pain diagnosis and the outpatient use of opioid medications, and Medicare patients < 65y were still significantly more likely to have a suboptimal bowel preparation. Because of the nature of the study, we were not able to determine the cause of disability for the Medicare < 65y patients.

Medicaid patients are also more likely to be taking opioid medications [15]. Again, even after controlling for a chronic pain diagnosis and the outpatient use of opioid medications, Medicaid/CICP patients were still more likely to have a suboptimal bowel preparation. Medicaid patients have been shown to have poorer health literacy than patients with private insurance [16]. Poor health literacy could influence the understanding of and compliance with bowel preparation instructions, leading to a suboptimal preparation. Health literacy should be investigated prospectively to delineate its contribution to suboptimal bowel preparation. Previous studies assessing the utility of a patient navigator for a screening colonoscopy program, including those with Medicaid, demonstrated improvements in screening rates and preparation quality [17, 18]. Due to the nature of this study, we were unable to analyze health literacy of the included patients.

The primary strengths of this study are the large sample size of patients, the adjustment for important covariates and confounders, and the clinically meaningful results. Another strength of this study was that both providers and patients did not know they were being studied. This increased the likelihood that providers followed their usual practice patterns. If providers knew they were being studied, it might influence their preparation quality ratings or their recommendation for an early repeat colonoscopy. Patients might be more adherent to the bowel preparation instructions if they knew they were in a study.

There are limitations to our study given its design. It is unknown at this point why the Medicare < 65y and Medicaid/CICP patient populations had such an increased likelihood of having a suboptimal bowel preparation. There may be other unmeasured confounders that could explain our findings (differences in race/ethnicity, smoking, and BMI). However, these variables are not incompletely captured or recorded for s substantial proportion of patients undergoing endoscopic procedures at UCH. There was also no inter-rater standardization of bowel preparation scores with the eight different attending endoscopists performing colonoscopies that were included in this study. However, all models were adjusted for the individual endoscopist. Assuming endoscopists have high intra-observer reliability in how they consistently rate a bowel preparation; the variation between endoscopists in the rating of bowel preparation quality would be accounted for as it relates to the primary and secondary outcomes. Even if there remains some variability between and within endoscopists on what is a “good” versus “fair” bowel preparation, our procedure time outcomes provide an objective measure that is consistent with Medicare < 65y and Medicaid/CICP populations being more likely to have a suboptimal bowel preparation, requiring additional cleaning time during the procedure. We did not have data on same-day cancellations or patients who did not present for their colonoscopy, which might reflect individuals who would have had a suboptimal preparation. Previous published data from a safety net hospital in the same metropolitan area as this study indicate that Medicaid/CICP and Medicare patients had significantly higher rates of nonattendance (and inadequate bowel preparations) [19]. Finally, there was a difference in the proportion of patients who used Moviprep^®^ compared to Colyte^®^ between groups, but the specific preparation had minimal effect on the outcomes and was accounted for in the statistical analyses. Despite these limitations, the magnitude of the increase in having a suboptimal bowel preparation and receiving a recommendation for an early repeat colonoscopy is striking for the Medicare < 65y and Medicaid/CICP patients. This is especially relevant in the Medicare < 65y patients where the ADR and AADR were significantly higher.

## Conclusion

We report the novel findings that Medicare < 65y and Medicaid patients are at increased risk of having a suboptimal bowel preparation and receiving a subsequent recommendation to have a screening or surveillance colonoscopy earlier than would otherwise be clinically indicated. We also found evidence that Medicare < 65y patients are more likely to have adenomas and advanced adenomas despite being more likely to have a suboptimal bowel preparation. Understanding why these groups are at increased risk for a suboptimal bowel preparation and designing interventions to reduce this rate has the potential to significantly improve patient outcomes and reduce healthcare costs.

## Abbreviations

ADR: Adenoma prevalence rate
AADR: advanced adenoma prevalence rate
CRC: colorectal cancer
CICP: Colorado Indigent Care Program
